# Deep brain stimulation response in obsessive–compulsive disorder is associated with preoperative nucleus accumbens volume

**DOI:** 10.1016/j.nicl.2021.102640

**Published:** 2021-03-22

**Authors:** Luka C. Liebrand, Paul Zhutovsky, Eva K. Tolmeijer, Ilse Graat, Nienke Vulink, Pelle de Koning, Martijn Figee, P. Richard Schuurman, Pepijn van den Munckhof, Matthan W.A. Caan, Damiaan Denys, Guido A. van Wingen

**Affiliations:** a Department of Psychiatry, Amsterdam UMC, University of Amsterdam, Amsterdam Neuroscience, Amsterdam, the Netherlands; b Department of Biomedical Engineering and Physics, Amsterdam UMC, University of Amsterdam, Amsterdam, the Netherlands; c Department of Clinical Psychology, VU University, Amsterdam Public Health Research, Amsterdam, the Netherlands; d Icahn School of Medicine at Mount Sinai, New York, NY, United States; e Department of Neurosurgery, Amsterdam UMC, University of Amsterdam, Amsterdam, the Netherlands; f Netherlands Institute for Neuroscience, Royal Academy of Arts and Sciences, Amsterdam, the Netherlands

**Keywords:** Deep brain stimulation, Obsessive–compulsive disorder, Nucleus accumbens, Anterior limb of the internal capsule, Treatment outcome prediction, Machine learning

## Abstract

**Background:**

Deep brain stimulation (DBS) is a new treatment option for patients with therapy-resistant obsessive–compulsive disorder (OCD). Approximately 60% of patients benefit from DBS, which might be improved if a biomarker could identify patients who are likely to respond. Therefore, we evaluated the use of preoperative structural magnetic resonance imaging (MRI) in predicting treatment outcome for OCD patients on the group- and individual-level.

**Methods:**

In this retrospective study, we analyzed preoperative MRI data of a large cohort of patients who received DBS for OCD (n = 57). We used voxel-based morphometry to investigate whether grey matter (GM) or white matter (WM) volume surrounding the DBS electrode (nucleus accumbens (NAc), anterior thalamic radiation), and whole-brain GM/WM volume were associated with OCD severity and response status at 12-month follow-up. In addition, we performed machine learning analyses to predict treatment outcome at an individual-level and evaluated its performance using cross-validation.

**Results:**

Larger preoperative left NAc volume was associated with lower OCD severity at 12-month follow-up (p_FWE_ < 0.05). None of the individual-level regression/classification analyses exceeded chance-level performance.

**Conclusions:**

These results provide evidence that patients with larger NAc volumes show a better response to DBS, indicating that DBS success is partly determined by individual differences in brain anatomy. However, the results also indicate that structural MRI data alone does not provide sufficient information to guide clinical decision making at an individual level yet.

## Highlights

• Preoperative MRI was associated with 12-months DBS treatment outcome in OCD patients.
• Larger nucleus accumbens volume was associated with larger clinical improvement.
• Machine learning analysis was not successful in predicting clinical improvement.

## 1 Introduction

Deep brain stimulation (DBS) is a new treatment option for approximately 10% of patients with obsessive–compulsive disorder (OCD) who do not benefit from conventional pharmacological and psychological therapies (Denys et al., 2020). On average, around 60% of these treatment-resistant patients respond to DBS (Alonso et al., 2015). Clinical predictors for DBS outcomes in OCD are scarce, with, e.g., an older age at onset of OCD being associated with better response on the group level (Alonso et al., 2015). However, these predictors cannot yet be used to determine which individual patients may or may not be suitable for DBS. While recent studies showed that treatment response might improve with diffusion magnetic resonance imaging (MRI) guided DBS targeting (Baldermann et al., 2019, Coenen et al., 2017, Liebrand et al., 2019), it is unlikely that all patients will become responders in the future. Since OCD has been associated with various structural brain abnormalities (Boedhoe et al., 2017, Gan et al., 2017, Hashimoto et al., 2014), differences in (individual) brain structure might be used to predict treatment response. Multiple studies used structural MRI data to predict treatment outcome in OCD (e.g., (Hashimoto et al., 2014, Yun et al., 2015)), but few studies examined neural biomarkers for treatment-resistant OCD (Dunlop et al., 2016, Van Laere et al., 2006). Nevertheless, the potential benefits of a reliable biomarker for DBS response are substantial. First, DBS is a long-term invasive treatment which carries several risks (Alonso et al., 2015, de Koning et al., 2011) and presents a possible burden to the patient, which could be avoided if potential non-responders are identified early. Second, DBS is a costly treatment with limited availability. Selecting only those patients who are likely to benefit would increase DBS’s cost-effectiveness, since the likelihood of DBS being cost-effective is only 57% over the first two years (Ooms et al., 2017). This could increase the availability of DBS, speeding up patients’ and referring clinicians’ decision to start treatment. In addition, an effective biomarker could provide valuable information regarding the pathophysiology of (treatment-resistant) OCD.

The nucleus accumbens (NAc) and the neighboring ventral capsule have been the most popular DBS targets for OCD (Alonso et al., 2015). These targets, which were adapted from white matter lesioning sites (Tierney et al., 2014), form a central hub within the cortico-striatal-thalamic-cortical (CSTC) loop (Wood and Ahmari, 2015). Previous findings suggest that DBS reduces OCD symptoms by disrupting pathological hyperconnectivity within the CSTC circuitry (Figee et al., 2013, Schmuckermair et al., 2013), preventing neurons in frontostriatal networks to synchronize (Bahramisharif et al., 2016, Smolders et al., 2013). The NAc is assumed to play an important role in integrating inputs within the CSTC circuitry, receiving dopaminergic and glutamatergic inputs from the ventral tegmental area and cortico-limbic regions, respectively (Wood and Ahmari, 2015). Successful DBS renormalizes abnormal striatal dopamine levels in OCD patients (Figee et al., 2014), which is in agreement with the assumed working mechanism of the same DBS target for depression (Coenen et al., 2011). Recent tractography studies further support the idea that connections to distal brain regions are important in DBS treatment response, even suggesting that white matter tracts running through the ventral capsule may be the optimal targets. Specifically, these studies have pointed towards the supero-lateral medial forebrain bundle (slMFB) (Coenen et al., 2018, Liebrand et al., 2019), possibly in combination with frontothalamic fibers (likely part of the anterior thalamic radiation (ATR)) (Baldermann et al., 2019). Complementary to their importance as targets, the NAc, slMFB and ATR might contain crucial information regarding treatment response.

In this retrospective study, we perform group- and individual-level analyses on preoperative structural MRI data to infer a potential relationship between voxel-wise grey- and white-matter volume (GM/WM) and DBS treatment response using one of the largest cohorts of OCD patients who received DBS to date. We hypothesized that grey matter (NAc) and white matter (ATR and slMFB) volume surrounding the DBS electrodes would be suitable for predicting improvement in OCD symptoms following DBS treatment. More exploratory, we also investigated DBS treatment effects on the whole-brain level.

## 2 Material and methods

### 2.1 Patients

We retrospectively retrieved and analyzed all available anonymized data of patients who received DBS for treatment-refractory OCD at the Amsterdam UMC (location AMC) in Amsterdam, The Netherlands, between 2005 and 2017. The first 16 patients participated in a clinical trial (Denys et al., 2010), while all consecutive patients received DBS as part of routine healthcare (Denys et al., 2020). We automatically retrieved preoperative MRI data of 63 patients. Data of six patients were excluded during preprocessing due to suboptimal segmentation or image artifacts (details in the *Imaging* section), so that datasets from 57 patients were used for the final analyses.

Patients aged 18–65 were eligible for treatment if they had a primary diagnosis of severe treatment-resistant OCD according to the DSM-IV (American Psychiatric Association, 2000) for over 5 years, with a minimum symptom score of 28 on the Yale-Brown obsessive compulsive scale (Y-BOCS). Patients were eligible for DBS if they did not previously respond to two 12-week trials with a selective serotonin reuptake inhibitors (SSRI) at maximum dosage, including augmentation with an atypical antipsychotic for 8 weeks, one 12-week trial of the maximum dosage clomipramine and cognitive behavioral therapy (CBT) at a center specialized in OCD (Denys et al., 2020). Contraindications for DBS were presence of psychotic disorders, recent substance abuse, and unstable neurological or coagulation disorders. Severe comorbid DSM diagnoses such as bipolar disorder or autism spectrum disorder were relative contraindications, outside of the first 16 patients included in our trial for whom these were always exclusion criteria. An independent psychiatrist monitored the inclusion process. More details about the included patients and inclusion and exclusion criteria can be found in (Denys et al., 2020).

Since this is a retrospective study with anonymized datasets that does not burden the patient, according to the Dutch Medical Research Involving Human Subjects Act (WMO) this study did not require approval from a medical-ethical committee. The institutional review board of Amsterdam UMC waived the obligation to obtain informed consent.

### 2.2 Treatment

#### 2.2.1 DBS lead implantation

Patients were bilaterally implanted under general anesthesia, according to standard stereotactic procedures. Surgical planning was performed based on anatomical landmarks in SurgiPlan (Elekta AB, Stockholm, Sweden), such that the active DBS contacts (model 3389, Medtronic, Minneapolis, US; 4x 1.5 mm contacts with 0.5 mm interspace) were placed in the ventral anterior limb of the internal capsule (ALIC) (van den Munckhof et al., 2013). The electrodes were coronally angled to follow the ALIC trajectory with an approximate anterior angle of 75°. Correct lead placement was ensured with co-registration of postoperative computed tomography (CT) to preoperative structural MRI.

#### 2.2.2 DBS optimization and CBT

The DBS device was switched on two weeks after surgery, marking the start of the optimization phase. In this phase, stimulation voltage, pulse duration and active contacts were subsequently updated in absence of clinical response. The clinical effect and tolerability of (side) effects of each new parameter combination was evaluated every two weeks, according to published protocols (van Westen et al., 2021) . The aim of DBS optimization was to find a clinically effective and tolerable parameter combination. Once achieved, these parameters were kept stable. The length of the optimization phase was not uniform, since the time to find the optimal stimulation parameters varied between patients. At the end of the optimization phase, patients received CBT during which they had to challenge their symptomatic behavior to augment the clinical effect of DBS (Mantione et al., 2014).

#### 2.2.3 Treatment outcome

Symptom severity was regularly assessed using the Y-BOCS, with a ≥35% symptom reduction with respect to the preoperative baseline determining treatment response. We computed DBS treatment response from baseline and 12-month follow-up Y-BOCS scores, which - outside of the first 16 patients - were obtained as part of routine clinical practice. In our analyses we first focused on the treatment response criterion as it has been used as a typical criterion of treatment success in DBS stimulation (Alonso et al., 2015). In addition, we also predicted the Y-BOCS score at 12-month follow-up directly as this approach should allow for better statistical modelling than prediction of (binarized) percentage change (Altman and Royston, 2006).

### 2.3 Imaging

#### 2.3.1 Data acquisition

The T1-weighted MRI data used in this study were all acquired for surgical planning according to clinical protocol. Given the large timeframe in which patients received DBS, different combinations of scanners/parameters were used in this study (Table S1).

#### 2.3.2 MRI preprocessing

Preoperative MRI data was preprocessed using the standardized pipeline of the CAT12 toolbox (r1450, http://www.neuro.uni-jena.de/cat) for SPM12 (v7487, https://www.fil.ion.ucl.ac.uk/spm/software/spm12) in the MATLAB programming language (R2018b, The Mathworks, Natick, MA). Preprocessing included inhomogeneity correction, partial volume based segmentation and spatial normalization to MNI space via Geodesic Shooting normalization (Ashburner and Friston, 2011) utilizing a template derived from 555 subjects of the IXI-database (http://brain-development.org/) provided by the CAT12 toolbox. The final GM/WM segmentations were modulated by the Jacobian determinant accounting for volume changes during the normalization process. The quality of the segmentations was investigated through the quality control options provided by the CAT12 toolbox and visual inspection. This led to the exclusion of five patients due to suboptimal segmentation quality and one patient due to an artifact in the original MRI scan. Finally, data were spatially smoothed with an 8 mm full-width-at-half-maximum kernel.

For the analyses whole-brain GM and WM masks were created by thresholding individual GM/WM images at 0.15 and only including voxels which survived thresholding across all patients. Bilateral ROI-specific masks for the NAc and the ATR were extracted from the subcortical Harvard-Oxford atlas (25% threshold of the maximum probability maps) and the JHU white-matter tractography atlas (25% threshold of the maximum probability maps), respectively, which are both included in the FSL library (Jenkinson et al., 2012). It is important to note that a large part of the slMFB is included in the atlas definition of the ATR.

We also calculated scalar momenta (Ashburner and Klöppel, 2011) as an additional and more advanced form of MRI data representation since a recent benchmarking study showed them to provide increased performance in pattern recognition tasks (Monté-Rubio et al., 2018). Details on their computation can be found in the Supplementary Methods.

### 2.4 Statistical analyses

#### 2.4.1 Clinical and demographic data

We summarized clinical and demographic data of the entire sample. To investigate whether responders and non-responders differed on demographic variables at baseline and follow-up (symptom severity) we used t-tests and Χ^2^-tests as appropriate. Tests were performed using the SPSS software (version 26).

#### 2.4.2 MRI Group-level analyses

All analyses were performed on ROI- (bilaterally) and whole-brain level. Group-differences between responders (n = 31) and non-responders (n = 26) were computed using the preprocessed and masked volume maps. Demeaned baseline Y-BOCS scores, age at baseline, sex, total intracranial volume (TIV), and scanner IDs (dummy-coded) were included as covariates in the analysis. The significance level was set at p < 0.05 family-wise error (FWE) corrected and estimated using the threshold-free cluster enhancement (TFCE) statistic with 10,000 permutations (Smith and Nichols, 2009). FWE corrections were performed using synchronized permutations and included corrections for all voxels within a mask/ROI, and two-sided tests (Alberton et al., 2020, Winkler et al., 2016). Additional multiple comparison corrections across two masks (NAc-ROI/ATR-ROI or GM/WM) were performed using Bonferroni-correction. All tests were performed using the PALM toolbox (a117, https://fsl.fmrib.ox.ac.uk/fsl/fslwiki/PALM).

Complementary to this analysis of group-differences, we performed group-level regression analyses between ROI/whole-brain segmentations and post-treatment Y-BOCS scores. We utilized the same covariates and statistical procedures as described above.

#### 2.4.3 MRI Individual-level analyses

In addition to group-level analyses, we also investigated the suitability of structural MRI for making individual-level predictions with machine learning procedures. For that we utilized linear-kernel support vector machine classification/regression (SVC/SVR) (Cortes and Vapnik, 1995, Drucker et al., 1997) and investigated its performance using 10-times-repeated-5-fold cross-validation (10x5 CV). In this procedure, the available data is randomly divided into 5 (approximately) equally sized folds, from which 4 folds are used as training data and the remaining 5th fold is used to estimate the performance of the SVC/SVR. This process is repeated five times, always using a different fold as the test set. The random assignment of data to folds is repeated ten times and performance across all 50 evaluations is averaged. This allows for an unbiased way to estimate generalization performance of machine learning models. Performance was measured as area-under-the-receiver-operator-curve (AUC), balanced accuracy, sensitivity and specificity in the classification case and as mean absolute error (MAE), mean squared error (MSE), root mean squared error (RMSE), Pearson correlation (r) and coefficient-of-determination (R^2^) for the regression case. We also applied label permutation tests (n = 1000) (Ojala and Garriga, 2010) to statistically determine whether the obtained performances (AUC for classification and MAE for regression) differed from chance-level at α = 0.05 Bonferroni-corrected for three tests corresponding to the different volumes per data scale (whole-brain or ROI). We corrected for three tests here because the individual-level analyses also considered the combination of each of our data representations (e.g., GM alone, WM alone and a combination of both GM + WM), contrary to the approach on the group-level.

We removed nuisance effects associated with age, sex, TIV, and scanner IDs via linear regression from the MRI data. Importantly, the estimation of the linear regression coefficients was always limited to the training set. In addition, baseline Y-BOCS score was added as a feature in both analyses. Given the high number of voxels in our dataset we implemented a feature selection approach. This corresponded to calculating Fisher scores (Li et al., 2017) in the classification case and Pearson correlations between each voxel and the Y-BOCS follow-up score across patients in the regression case. These calculations were again only performed on the training set. To determine the optimal percentage of features to select, a nested cross-validation procedure (with 5-fold CV as the inner CV) was implemented. All analyses were run for whole-brain GM/WM and NAc/ATR ROIs and the combination of GM/WM and NAc/ATR data. The combination corresponded to just concatenating the different feature maps. In addition, we also repeated the analysis for scalar momenta as a more advanced form of data representation. All analyses were implemented in the Python programming language (3.7.6) utilizing the scikit-learn toolbox (0.22.1, (Pedregosa et al., 2011)).

## 3 Results

### 3.1 Clinical and Demographic Data

A summary of the clinical and demographic data and statistical tests between responders and non-responders are reported in Table 1. Responders and non-responders did not statistically differ at baseline; only Y-BOCS scores at 12-month follow-up differed significantly between these groups (t(49.571) = -9.986, p < 0.001, see Figure S1 for trajectories of Y-BOCS scores per patient).

**Table 1:** Demographic and clinical variables (mean (SD) [range]).

	All (n = 57)	Responder (n = 31)	Non-Responder (n = 26)	
Age [years]	42.65 (11.17) [23–69]	43.48 (11.10) [30–69]	41.65 (11.40) [23–65]	t(55) = 0.612, p = 0.543^a^
Sex (F/M)	41/16	21/10	20/6	χ^2^(1) = 0.590, p = 0.442^b^
Age at onset of OCD [years]	16.23 (9.13) [4–52]	17.45 (8.89) [6–52]	14.77 (9.38) [4–40]	t(55) = 1.107, p = 0.273^a^
Duration of illness [years]	26.82 (10.69) [7–51]	26.61 (9.65) [12–51]	27.08 (12.00) [7–50]	t(55) = -0.162, p = 0.872^a^
MRI sequence^c^	6/24/5/15/7	3/14/2/7/5	3/10/3/8/2	χ^2^(4) = 1.794, p = 0.774^b^
TIV [ml]	1359.82 (156.65) [1102.17–1795.69]	1358.00 (159.13) [1129.47–1795.69]	1361.99 (156.76) [1102.17–1637.40]	t(55) = -0.095, p = 0.925^a^
Baseline Symptom Severity:				
Y-BOCS	33.85 (3.22) [28–40]	33.94 (3.25) [28–40]	33.77 (3.24) [28–40]	t(55) = 0.193, p = 0.848^a^
HAM-A	26.65 (7.96) [11–45]	26.68 (8.22) [11–42]	26.62 (7.80) [12–45]	t(55) = 0.029, p = 0.977^a^
HAM-D	21.38 (5.89) [8–35]	21.30 (6.18) [11–31]	21.46 (5.67) [8–35]	t(54) = -0.101, p = 0.920^a^
Baseline medication:				χ^2^(5) = 8.954, p = 0.111^b^
None	7	4	3	
SSRI	13	5	8	
SSRI + antipsychotic	14	11	3	
Clomipramine	6	1	5	
Clomipramine + antipsychotic	16	9	7	
Other	1	1	0	
Baseline Comorbidities:				
Mood Disorders	30	17	13	χ^2^(1) = 0.133, p = 0.716^b^
Anxiety Disorders	6	2	4	χ^2^(1) = 1.198, p = 0.274^b^
Addiction	3	2	1	χ^2^(1) = 0.193, p = 0.661^b^
Eating Disorders	4	3	1	χ^2^(1) = 0.795, p = 0.373^b^
Personality Disorders	11	6	5	χ^2^(1) < 0.001, p = 0.991^b^
Other	3	1	2	χ^2^(1) = 0.566, p = 0.452^b^
Stimulation settings^d^:				
# Active contacts (1/2/3/4)	1/39/6/4 *3 unknown*	1/23/1/1 *3 unknown*	0/16/5/3	χ^2^(3) = 4.00, p = 0.26
Voltage (V)	4.32 (0.90) [2.80–6.30]	4.28 (0.87) [3.00–6.30]	4.35 (0.95) [2.80–6.00]	t(51) = 0.285, p = 0.77
Pulse width (µs)(60/90/120/150/180)	3/37/4/4/2 *3 unknown*	3/19/2/2/2 *1 unknown*	0/18/2/2/0 *2 unknown*	χ^2^(4) = 6.667, p = 0.15
Frequency (Hz)(130/180/185)	37/2/11 *3 unknown*	22/1/5 *1 unknown*	15/1/6 *2 unknown*	χ^2^(3) = 6.00, p = 0.20
Post-treatment Symptom Severity:				
Y-BOCS	19.35 (9.40) [0–35]	12.61 (6.94) [0–24]	27.38 (4.06) [22–35]	t(49.571) = -9.986, p < 0.001^e^, *

F: Female; M: Male; MRI: Magnetic Resonance Imaging; TIV: Total Intracranial Volume; Y-BOCS: Yale-Brown Obsessive Compulsive Scale; HAM-A: Hamilton Anxiety Rating Scale; HAM-D: Hamilton Depression Rating Scale; SSRI: Selective Serotonin Reuptake Inhibitor.

a independent samples *t*-test - equal variances assumed.

b Chi-square test.

c MRI sequence correspond to the five different MRI scanners/sequences described in the supplementary materials.

d Stimulation settings for n = 53, data of 4 patients (2 responders) were not automatically retrievable.

e independent samples *t*-test - equal variances not assumed.

* p < 0.05.

### 3.2 MRI Group-level analyses

**Fig. 1:**
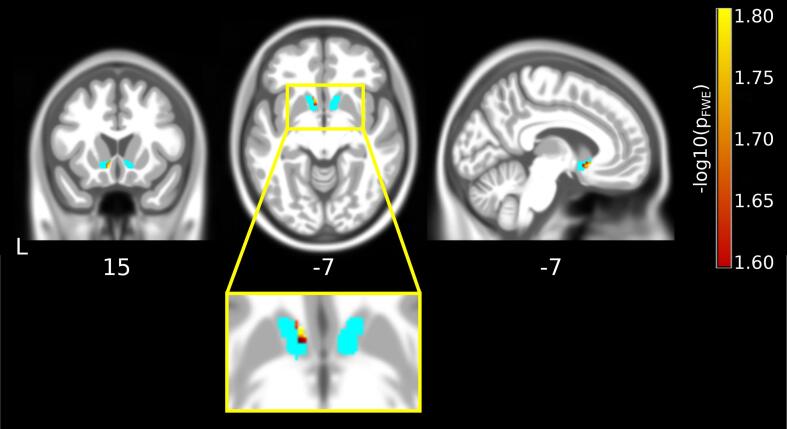
Negative group-level association between the post-treatment Y-BOCS score and pre-treatment grey-matter volume of voxels within the nucleus accumbens ROI (in cyan). Results are overlaid over the average T1 image calculated from 555 subjects of the IXI database provided by the CAT12 toolbox. Coordinates are given in MNI space. Significant associations are shown in hot colors thresholded at p_FWE_ < 0.025 (-log10(0.025) = 1.602). (For interpretation of the references to colour in this figure legend, the reader is referred to the web version of this article.)

**Fig. 2:**
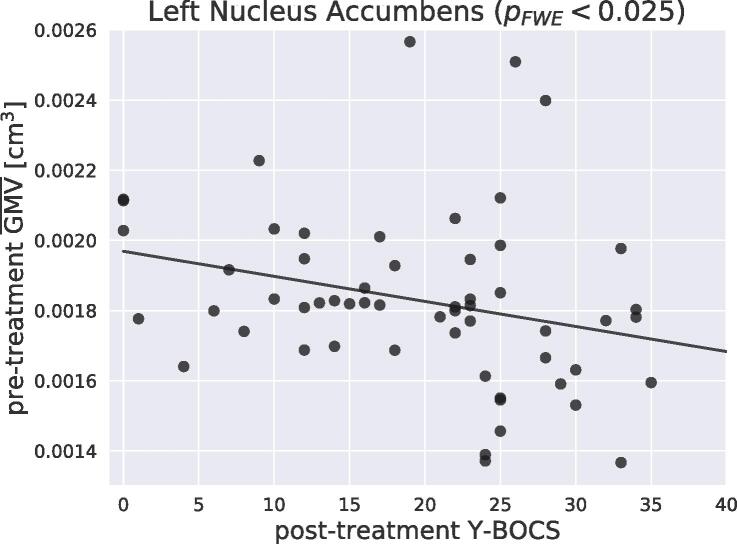
Negative group-level association between post-treatment Y-BOCS and the average grey-matter volume of all significant voxels within the left nucleus accumbens ROI.

The results of the group-level analyses are summarized in Fig. 1 and Fig. 2. We found a significant association between Y-BOCS scores at 12-month follow-up and left NAc grey-matter volume (31 voxels, maximum: −7.5, 15, −7.5 [mm], TFCE-value: 85.91) (Fig. 1). Lower follow-up Y-BOCS scores were associated with larger preoperative grey-matter volume (Fig. 2). This result remained significant when an additional covariate encoding time since first DBS operation (mean-centered) performed in the patient sample was added to the model. We did not find significant associations between clinical outcomes and volumes of the right NAc, or ATR. However, right NAc grey-matter volume did show a comparable association at the uncorrected level, implying a potential lack of power to detect an effect. When comparing groups, larger NAc grey-matter volume in the same voxels was trend-level significant for responders over non-responders. There were no significant associations in the exploratory whole-brain analyses.

### 3.3 MRI Individual-level analyses

Results of individual-level regression and classification analyses are reported in Table 2, Table 3. None of the MRI data representations exceeded chance-level performance neither for the regression nor the classification analysis, neither when using whole-brain or ROI data.

**Table 2:** Classification performance for different MRI data representations. Estimated using 10-times repeated 5-fold cross-validation (mean (SD) [range]).

	AUC	Accuracy [%]	Sensitivity [%]	Specificity [%]
GM	0.468 (0.124) [0.167–0.694]	48.21 (10.25) [26.67–72.86]	56.76 (19.96) [16.67–100]	39.67 (19.57) [0–80]
WM	0.422 (0.173) [0–0.80]	44.90 (14.52) [16.67–72.86]	57.52 (23.27) [0–100]	32.27 (21.58) [0–60]
GM + WM	0.433 (0.171) [0–0.833]	48.10 (15.56) [0–80.00]	60.67 (22.71) [0–100]	35.53 (19.40) [0–66.67]
NAc	0.543 (0.213) [0.067–0.933]	57.30 (13.23) [16.67–83.33]	62.19 (19.62) [16.67–100]	52.40 (20.00) [0–100]
ATR	0.509 (0.201) [0.10–0.933]	42.86 (14.86) [16.67–73.33]	47.19 (19.16) [0–85.71]	38.53 (24.02) [0–100]
NAc + ATR	0.481 (0.162) [0.133–0.767]	48.57 (14.57) [16.67–75.71]	54.14 (17.65) [16.67–85.71]	43.00 (22.41) [0–80]
Scalar Momentum	0.546 (0.136) [0.233–0.857]	51.52 (10.24) [33.33–75.71]	56.38 (17.21) [16.67–85.71]	46.67 (18.52) [0–100]

AUC: Area-under-receiver-operator-curve; GM: Grey-matter volume; WM: White-matter volume; NAc: Grey-matter volume of Nucleus Accumbens; ATR: White-matter volume of Anterior Thalamic Radiation

**Table 3:** Regression performance for different MRI data representations. Estimated using 10-times repeated 5-fold cross-validation (mean (SD) [range]).

	MAE	MSE	RMSE	Pearson correlation	R^2^
GM	9.23 (2.03) [5.37–13.85]	129.44 (47.16) [50.35–247.44]	11.19 (2.05) [2.05–15.73]	−0.036 (0.012) [−0.496–0.156]	−0.684 (0.572) [−2.556–0.156]
WM	9.07 (1.64) [5.91–13.04]	123.87 (44.80) [51.24–278.98]	10.97 (1.90) [7.16–16.70]	0.012 (0.246) [−0.487–0.549]	−0.628 (0.568) [−3.009–0.253]
GM + WM	8.80 (1.73) [5.35–13.00]	119.17 (40.90) [50.91–238.46]	10.77 (1.82) [7.14–15.44]	0.055 (0.247) [−0.519–0.446]	−0.592 (0.655) [−2.812–0.157]
NAc	7.84 (1.46) [4.89–11.81]	92.87 (32.02) [38.43–178.91]	9.50 (1.61) [6.20–13.38]	0.365 (0.272) [−0.208–0.738]	−0.253 (0.514) [−1.477–0.430]
ATR	9.02 (1.93) [5.44–16.01]	128.17 (53.35) [54.08–329.40]	11.11 (2.20) [7.35–18.15]	0.133 (0.279) [−0.662–0.583]	−0.694 (0.686) [−2.910–0.305]
NAc + ATR	9.17 (1.72) [4.43–13.57]	126.27 (43.82) [33.90–237.21]	11.07 (1.98) [5.82–15.40]	0.253 (0.340) [−0.552–0.772]	−0.698 (0.672) [−2.443–0.471]
Scalar Momentum	8.96 (1.99) [5.59–17.74]	119.07 (58.20) [55.35–437.43]	10.69 (2.20) [7.44–20.91]	0.161 (0.293) [−0.585–0.719]	−0.586 (0.829) [−5.286–0.305]

MAE: Mean absolute error; MSE: Mean squared error; RMSE: Root mean squared error; R^2^: Coefficient of determination; GM: Grey-matter volume; WM: White-matter volume; NAc: Grey-matter volume of Nucleus Accumbens; ATR: White-matter volume of Anterior Thalamic Radiation

## 4 Discussion

The aim of this study was to investigate the relationship between voxel-wise brain volumetry and DBS treatment response in OCD. We related the 12-month follow-up Y-BOCS score to volumetric differences on the group-level, and tested whether brain volumes were predictive of outcomes on an individual-level with SVC/SVR. In our sample we found that larger preoperative volumes of the left NAc were significantly associated with lower Y-BOCS scores at 12-month follow-up on the group-level. However, our machine learning analyses did not generate models that could predict individual-level outcome above chance-level.

The NAc is involved in the pathophysiology of OCD and is centrally located in the CSTC circuitry. For this reason the NAc was our original DBS target (Denys et al., 2010), although the clinically effective contacts were located in the ventral ALIC white matter just above the NAc (van den Munckhof et al., 2013). Given the suspected disruptive effect of DBS on connectivity (Bahramisharif et al., 2016, Smolders et al., 2013), it may be remarkable that larger NAc volumes are associated with better outcomes. Potentially, the beneficial effect of stimulation is larger in patients with an increased NAc volume, meaning that patients with smaller NAc volumes could be even more treatment-resistant*.* Previous studies suggest that treatment outcome depends on proximity of stimulation to white matter bundles in the vALIC (Baldermann et al., 2019, Liebrand et al., 2019). It is possible that larger NAc volumes led to electrode positioning such that stimulation was closer to the relevant white matter structures. In this case, larger NAc volumes would rather reflect better odds of achieving optimal electrode positioning than directly predict response. Given the respective scales of the white matter variability in the vALIC (Liebrand et al., 2019) and NAc volumetric differences, the chance that these relatively small volumetric differences were responsible for a difference in electrode positioning large enough to affect treatment outcome appears small. Conversely, the surgical methods are unlikely to have played a role in the observed asymmetry. Although the left-sided electrode was usually implanted first, followed by the right-sided electrode since the infra-clavicular stimulator is usually implanted on the right side, the time between the two electrode insertions is short. Measures like glue in the burr-holes prevent the leakage of cerebrospinal fluid and intraoperative brain shift hardly occurs any longer so there is no asymmetry to be expected in the potential targeting inaccuracy.

Research into the role of the NAc in the context of predicting treatment outcome has been scarce, but earlier studies have linked the NAc to pharmacotherapy and psychotherapy resistance. Treatment-resistant OCD patients showed hypo-responsivity of the NAc during the anticipation of rewards (Figee et al., 2011), as well as micro-structural alterations of the NAc as measured with diffusion tensor fractional anisotropy (Li et al., 2014). More recently, a study investigating patients who received treatment with CBT and SSRIs for anxiety disorders found that larger baseline NAc volumes were associated with a larger reduction of anxiety symptoms (Burkhouse et al., 2020). The authors suggested that treatments targeting anxiety-related avoidance behavior were more effective in patients with larger pretreatment deficits in the systems responsible for the avoidance behavior, which was supported by studies showing a relationship between larger baseline NAc volume and more severe anxiety symptoms (Günther et al., 2018, Kühn et al., 2011) as well as between NAc structural alterations and avoidance behaviors in patients with anxiety symptoms (Lago et al., 2017). In our experience, the treatment effect in DBS for OCD is achieved by an initial anxiolytic effect that is further augmented by exposure-based CBT (Denys et al., 2010, Mantione et al., 2014). Taken together, the larger reduction of OCD symptoms in patients with larger NAc volumes may result from a larger effect of DBS on avoidance behaviors, which needs to be tested in future research. Patients with smaller NAc volumes might benefit less from treatment because the NAc’s ability to integrate dopaminergic inputs during reward processing may be impaired, which could interfere with resumption of normal functioning within the CSTC circuitry.

While an association between the left NAc volume and follow-up Y-BOCS was found on the group-level, both the SVC/SVR approaches did not yield predictive values significantly above chance-level. One possible explanation is that multivariate analyses typically require larger samples than univariate analyses (Bzdok and Ioannidis, 2019). Our sample, while sizable for psychiatric DBS studies, may not have been large enough to detect the complex patterns needed for individual-level prediction. Given the group-level association between NAc volume and Y-BOCS follow-up score, it is possible that future studies with larger sample sizes may be able to find a structural MRI biomarker. However, another possibility is that NAc volume differences may be obscured by variation in OCD subtypes, or that subcortical alterations in OCD may depend on gender (Zhang et al., 2019). Development of models that account for these subgroups requires a further increase in sample size.

Given that structural alterations in OCD patients are small, often finding limited effect sizes in large multicenter group studies (Bruin et al., 2020), inclusion of additional imaging modalities, such as (resting-state) functional and diffusion MRI, could strengthen the predictive value of our models (van Waarde et al., 2015, Zhutovsky et al., 2019). The availability of functional and diffusion MRI scans for our cohort was limited, since most patients were routinely treated and not enrolled into a neuroimaging study. Conversely, structural MRI scans were readily available due to their necessity in surgical planning. Increased use of tractography in surgical planning for DBS for OCD will improve future availability of diffusion MRI scans (Baldermann et al., 2019, Coenen et al., 2017, Liebrand et al., 2019).

### 4.1 Limitations

The most notable limitation to this study is its sample size. Despite being among the largest studies on patients with DBS for psychiatric conditions, our sample was still modest for state-of-the-art machine learning applications. This could have caused our individual-level prediction to be underpowered. In addition, at these sample sizes it is impossible to stratify for differences in disease history, medication use, and OCD subtypes. Given the long timeframe during which this dataset was acquired, the only possibility of rapidly increasing the number of patients would be pooling data from different sites. However, pooling data across centers comes with its own set of challenges, like variation in diagnosis and inclusion criteria per institute, non-uniformity of stimulation targets and parameters across sites, and restrictions on data sharing due to privacy laws. These challenges might explain the lack of large retrospective multicenter studies in DBS for OCD.

Another limitation lies in the naturalistic follow-up for all patients after the first 16 patients. After the initial trial (Denys et al., 2010) DBS was approved for routine care for treatment-refractory OCD. Combined with the long inclusion period, this caused the treatment follow-up and imaging parameters to vary over time. To address this issue, we corrected for scanner/parameter combinations in our analyses. More importantly, after analysis of our clinical trial cohort showed that active contacts were always located in the vALIC (van den Munckhof et al., 2013), the targeting was altered so that the 3 topmost contacts were always placed in the vALIC white matter for new patients. This heterogeneity in targeting strategies potentially confounded our results, although given the large degree of overlap in anatomical positioning of the active contacts in our previous study on a subsample of this dataset (Liebrand et al., 2019) we expect no systematic differences between (non–)responders. The more important relationship between positioning of the electrodes and individual white matter connections is impossible to ascertain without additional diffusion MRI data. Comparisons over such a long timeframe could have been improved by using a fixed protocol. However, it is debatable whether this would have been beneficial for the patients. Patients should be able to benefit from new insights gained with experience. We attempted to address this limitation by including a covariate indicating the time since first operation into our model which showed that the association remained significant. However, it is important to note that changes in targeting cannot be assumed to vary linearly across patients and such an adjustment cannot circumvent the need for a fixed protocol.

The current study focused on response to DBS within a 12-month follow-up. This time period enables the identification of most responders, though a small minority only starts responding between one to two years after the application of DBS (van Westen et al., 2021). Therefore, future studies could investigate whether the observed associations also applies to patients with a delayed response.

### 4.2 Conclusions

We performed the – to our knowledge – largest neuroimaging study on patients who received DBS for treatment-refractory OCD. Our results showed that increased left-side NAc volume was associated with a lower 12-month follow-up Y-BOCS score. Caveated by non-significant predictions at the individual-level, group-level associations between NAc volume and DBS treatment outcomes suggest that patients with a larger NAc are better able to benefit from CBT and regain their functioning after receiving DBS. Although individual-level predictions with SVC/SVR were not predictive, the results could provide a stepping stone for future biomarker studies for DBS for OCD. It is our hope that these studies will contribute to improved informing and supporting of patients and clinicians in their decision-making process, which can help optimize the response rate, reduce potential harm or burden to patients, and improve the allocation of resources.

## CRediT authorship contribution statement

**Luka C. Liebrand:** Conceptualization, Methodology, Formal analysis, Data curation, Writing - original draft. **Paul Zhutovsky:** Methodology, Software, Formal analysis, Writing - original draft. **Eva K. Tolmeijer:** Data curation. **Ilse Graat:** Data curation. **Nienke Vulink:** Investigation. **Pelle de Koning:** Investigation. **Martijn Figee:** Investigation. **P. Richard Schuurman:** Investigation. **Pepijn van den Munckhof:** Investigation. **Matthan W.A. Caan:** Supervision. **Damiaan Denys:** Funding acquisition. **Guido A. van Wingen:** Conceptualization, Supervision, Funding acquisition.

## Declaration of Competing Interest

The authors declare that they have no known competing financial interests or personal relationships that could have appeared to influence the work reported in this paper.
