# Supplementary material for: Deep brain stimulation response in obsessive–compulsive disorder is associated with preoperative nucleus accumbens volume
Source: Neuroimage Clin. 2021 Mar 22;30:102640. doi: 10.1016/j.nicl.2021.102640 (PMC8044711; doi:10.1016/j.nicl.2021.102640)
Supplement: Supplementary data 1 [file mmc1.docx]

**Supplemental Information**

**Luka C. Liebrand^1,2,^***, **Paul Zhutovsky^1,^***, Eva K. Tolmeijer^3^, Ilse Graat^1^, Nienke Vulink^1^, Pelle de Koning^1^, Martijn Figee^1,4^, P. Richard Schuurman^5^, Pepijn van den Munckhof^5^, Matthan W.A. Caan^2^, Damiaan Denys^1,6^, Guido A. van Wingen^1
1^ Department of Psychiatry, Amsterdam UMC, University of Amsterdam, Amsterdam Neuroscience, Amsterdam, The Netherlands
^2^ Department of Biomedical Engineering and Physics , Amsterdam UMC, University of Amsterdam, , Amsterdam, The Netherlands
^3^ Department of Clinical Psychology, VU University, Amsterdam Public Health Research, Amsterdam, The Netherlands
^4^ Icahn School of Medicine at Mount Sinai, New York, NY, United States
^5^ Department of Neurosurgery, Amsterdam UMC, University of Amsterdam, Amsterdam, The Netherlands
^6^ Netherlands Institute for Neuroscience, Royal Academy of Arts and Sciences, Amsterdam, Netherlands
*** Both authors contributed equally to this work.**

**Calculation of Scalar Momenta**

In addition to the calculation of the grey- and white-matter volumes (GM/WM) as presented in the main manuscript we also investigated scalar momenta (Ashburner and Klöppel, 2011) as an additional structural MRI representation for our individual-level classification/regression analyses. The reason for this was a recent benchmarking study which showed that scalar momenta outperformed many of the other VBM types of data representations for multiple datasets and various different clinical and non-clinical tasks (Monté-Rubio et al., 2018). The calculation of the scalar momenta followed the corresponding description provided in the benchmarking paper and included: 1. Use of SPM12’s unified segmentation (Ashburner and Friston, 2005) approach to obtain GM, WM and cerebrospinal fluid tissue types for each individual MRI scan. The segmentation was set up according to the VBM tutorial by John Ashburner (<http://www.fil.ion.ucl.ac.uk/~john/misc/VBMclass10.pdf>), 2. Normalization of the GM and WM segmentations to MNI space by using the Geodesic Shooting Toolbox for SPM12 (Ashburner and Friston, 2011), utilizing the same group template derived from 555 subjects of the IXI-database (<http://brain-development.org/>) as provided by the CAT12 toolbox which was also considered for the main analyses, 3. Calculation of scalar momenta using the Geodesic Shooting Toolbox, 4. Spatial smoothing with a 12 mm^3^ full-width at half-maximum kernel as it was shown to provide the best performance in the benchmarking study. Scalar momenta are computed by scaling the difference between the template GM/WM segmentations and their corresponding warped individual segmentations with the Jacobian-determinant. They can therefore be seen as Jacobian-scaled residual-errors of the normalization of the individual segmentations. In accordance, with (Monté-Rubio et al., 2018) we considered the scalar momenta for GM and WM together in our analyses.

**Table S1.** Scanning parameters for the different scans. The order of the labels (A/B/C/D/E) correspond to the labels used in the main text and Table 1.

| Sequence Label | Vendor & Scanner name | Field Strength (T) | Acquisition matrix | Acquisition resolution (mm^3^) | Repetition time (ms) | Echo time (ms) | Flip angle (°) |
| --- | --- | --- | --- | --- | --- | --- | --- |
| A | Philips Intera | 3 | 256x256 | 1x1x1 | 9 | 4 | 8 |
| B | Philips Ingenia | 3 | 284x284 | 0.9x0.9x0.9 | 9 | 4 | 8 |
| C | Siemens Avanto | 1.5 | 256x256 | 1x1x1 | 1900 | 3 | 15 |
| D | Genesis Signa | 1.5 | 256x256 | 1x1x1 | 13 | 6 | 20 |
| E | Philips Intera | 3 | 256x256 | 0.88x0.88x1.2 | 10 | 5 | 8 |

**
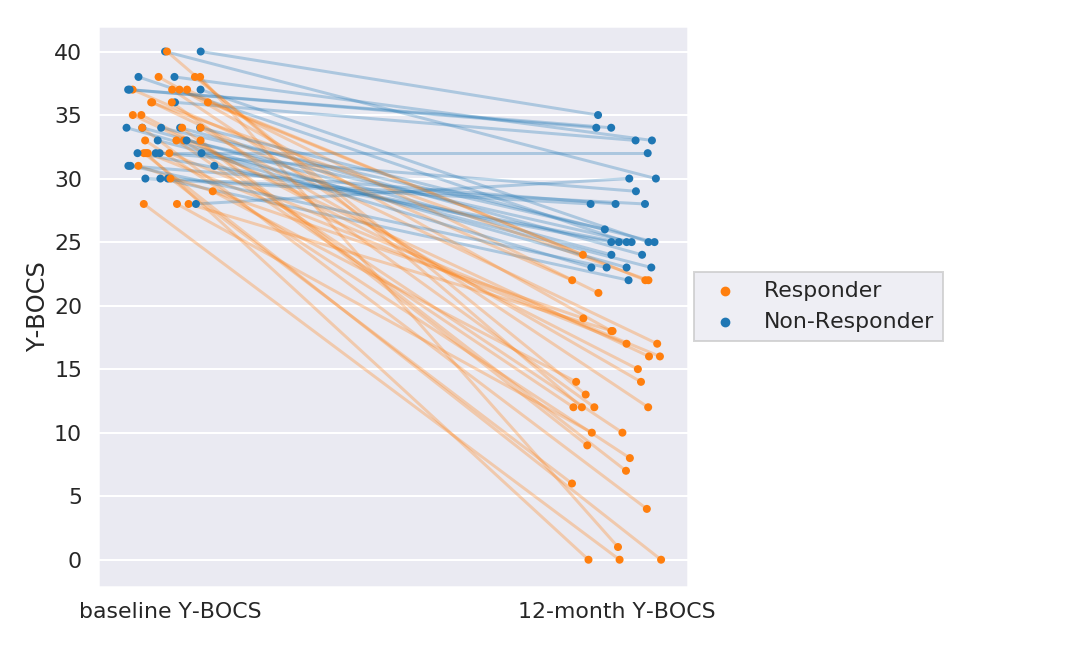
**

**Figure S1:** Trajectories of baseline to 12-month follow-up progression of the Yale-Brown obsessive compulsive scale (Y-BOCS) total score for each individual patient included in this study. Each dot represents a patient, color coded for whether the baseline Y-BOCS score decreased by at least 35% from baseline to 12-month follow-up (Responder) or not (Non-Responder). Lines connect data of the same patient.

**References**

Ashburner, J., Friston, K.J., 2011. Diffeomorphic registration using geodesic shooting and Gauss–Newton optimisation. Neuroimage 55, 954–967. https://doi.org/10.1016/j.neuroimage.2010.12.049

Ashburner, J., Friston, K.J., 2005. Unified segmentation. Neuroimage 26, 839–851. https://doi.org/https://doi.org/10.1016/j.neuroimage.2005.02.018

Ashburner, J., Klöppel, S., 2011. Multivariate models of inter-subject anatomical variability. Neuroimage 56, 422–439. https://doi.org/10.1016/j.neuroimage.2010.03.059

Monté-Rubio, G.C., Falcón, C., Pomarol-Clotet, E., Ashburner, J., 2018. A comparison of various MRI feature types for characterizing whole brain anatomical differences using linear pattern recognition methods. Neuroimage 178, 753–768. https://doi.org/10.1016/j.neuroimage.2018.05.065
